# Editorial: Mathematical modeling of medication nonadherence

**DOI:** 10.3389/fphar.2025.1741041

**Published:** 2025-11-26

**Authors:** Sean D. Lawley, Teresa B. Gibson, Zheng Jiao

**Affiliations:** 1 Department of Mathematics, University of Utah, Salt Lake City, UT, United States; 2 School of Mathematics and Statistics, Rochester Institute of Technology, Rochester, NY, United States; 3 Shanghai Chest Hospital, Shanghai Jiao Tong University, School of Medicine, Shanghai, China

**Keywords:** adherence, nonadherence, mathematical modeling, stochastic modeling, statistical analysis, artificial intelligence

Medication adherence is the process by which patients take their medications as prescribed (Vrijens et al., 2012). Medication *non*adherence is an age-old problem, as even Hippocrates warned physicians to “keep a watch also on the faults of the patients, which often make them lie about the taking of things prescribed” (Potter et al., 1923). Today, medication nonadherence results in over 100,000 preventable deaths and more than $100 billion in healthcare costs each year in the United States alone (Osterberg and Blaschke, 2005; Kini and Ho, 2018). In fact, the World Health Organization has claimed (Sabaté et al., 2003):

“Increasing the effectiveness of adherence interventions may have a far greater impact on the health of the population than any improvement in specific medical treatments.”

A former United States surgeon general famously observed, “drugs do not work in patients who do not take them” Lindenfeld and Jessup, 2017).

Medication nonadherence is challenging to understand and mitigate for many reasons. For one, nonadherence can be erratic, as patients do not miss doses on neat, predictable schedules. Indeed, adherence data for an individual patient over time shows doses taken on-time, doses taken late, doses skipped, double doses, etc. (Vrijens et al., 2008). Furthermore, these seemingly stochastic, temporal fluctuations in adherence vary considerably between patients (Blaschke et al., 2012), with perhaps at least six qualitatively distinct patterns seen in different patients (Urquhart, 2002). In addition to temporal and patient heterogeneity, the physiological consequences of nonadherence can vary considerably between drugs. For example, missing a morning dose of one medication may go largely undetected by the patient or might entail a lethargic afternoon, but a missing a dose of an antiepileptic drug might cause a seizure (Pellock et al., 2004). Moreover, even within a specific drug class, some drugs maintain efficacy despite lapses in adherence (so-called “forgiving” drugs), whereas other drugs require nearly perfect adherence to be effective (Osterberg et al., 2010).

Due to this complexity, mathematical and computational approaches are emerging as powerful tools to combat medication nonadherence. For instance, stochastic analysis can leverage the science of pharmacometrics to investigate remedial dosing protocols and design regimens to mitigate nonadherence (Jun and Nekka, 2007; Chen et al., 2013; Gu et al., 2020; Counterman and Lawley, 2021; Counterman and Lawley, 2022; Dai et al., 2025; Liu et al., 2024; Li et al., 2023; Clark and Lawley, 2022; Clark and Lawley, 2024). Furthermore, “drug forgiveness” has been quantified with a number of approaches (Urquhart, 1997; Nony et al., 2002; Boissel and Nony, 2002; Goue Gohore et al., 2010; Dartois, 2011; Lowy et al., 2011; Assawasuwannakit et al., 2015; Assawasuwannakit et al., 2016; Pellock and Brittain, 2016; Morrison et al., 2017; McAllister and Lawley, 2022; Hwai-Ray and Lawley, 2024; Cengiz et al., 2025; Tung and Lawley, 2025), which enables the identification and design of drugs which are robust to nonadherence. In effect, the mathematical equations are a laboratory to conduct experiments which would not be feasible in human trials, such as quantifying how therapeutic efficacy depends on adherence rates and patterns. In addition, methods employing artificial intelligence (AI) and sophisticated statistical approaches are being applied to swaths of adherence data to predict, detect, and ameliorate nonadherence (Babel et al., 2021; Bohlmann et al., 2021; Gibson, 2022; DeClercq and Choi, 2020; Haff et al., 2022).

This Research Topic presents five articles in this burgeoning field. Three of the articles involve AI methods. Chang et al. propose algorithms based on machine learning to predict nonadherence and suggest dynamic, personalized interventions. Xie proposes an intelligent computing framework via a hierarchical latent prior structure to support pharmacotherapy decision-making by predicting adherence patterns and clinical outcomes for individual patients. Reis et al. review 7 studies of AI-based tools designed to support patient medication use and highlight their promise for improving medication adherence. Together, these three articles highlight the promise of AI for alleviating the deleterious health effects of nonadherence. Leguizamo-Martinez et al. employ group-based statistical methodologies to identify adherence patterns over time. These authors use prescription data on nearly 30,000 patients who initiated direct oral anticoagulant therapy to categorize adherence trajectories and quantify how various factors correlate with nonadherence. Yan et al. created and administered a questionnaire to evaluate factors influencing the adherence of asthmatic children to inhaled corticosteroids. The authors then employ a variety of statistical tests to evaluate their questionnaire and its results, and ultimately obtain quantitative estimates of how various factors affect adherence in this patient population.

The articles in this Research Topic demonstrate the significant potential of mathematical modeling and AI in tackling the heterogeneous challenge of medication nonadherence. These emerging approaches are driving a paradigm shift from static assessment toward predictive, dynamic frameworks that can mitigate nonadherent behaviors. Looking ahead, the imperative is to accelerate the translation of these computational tools from theory into clinical practice, enabling the design of more “forgiving” pharmacotherapies and the delivery of personalized interventions. Fulfilling this potential is critical to realizing the profound impact that improved adherence interventions can have on population health, as emphasized by the World Health Organization.
